# An Optimization Procedure for Preparing Aqueous CAR/HP-CD Aggregate Dispersions

**DOI:** 10.3390/molecules26247562

**Published:** 2021-12-13

**Authors:** Enrika Celitan, Ruta Gruskiene, Jolanta Sereikaite

**Affiliations:** Department of Chemistry and Bioengineering, Vilnius Gediminas Technical University, LT-10223 Vilnius, Lithuania; enrika.celitan@gmc.vu.lt (E.C.); ruta.gruskiene@vgtu.lt (R.G.)

**Keywords:** β-carotene, 2-hydroxypropyl-β-cyclodextrin, aggregate, optimization of procedure, co-precipitation

## Abstract

β-Carotene is a very important molecule for human health. It finds a large application in the food industry, especially for the development of functional foods and dietary supplements. However, β-carotene is an unstable compound and is sensitive to light, temperature, and oxygen. To overcome those limitations, various delivery systems were developed. The inclusion of β-carotene by cyclodextrin aggregates is attractive due to non-toxicity, low hygroscopicity, stability, and the inexpensiveness of cyclodextrins. In this study, β-carotene/2-hydroxypropyl-β-cyclodextrin aggregates were prepared based on the procedure of the addition of β-carotene in an organic solvent to the hot water dispersion of 2-hydroxypropyl-β-cyclodextrin and the following instant evaporation of the organic solvent. The best conditions for the aggregate preparation were found to be as follows: 25% concentration of 2-hydroxypropyl-β-cyclodextrin in water, 65 °C temperature, and acetone for β-carotene dissolution. The efficiency of entrapping was equal to 88%. The procedure is attractive due to the short time of the aggregate preparation.

## 1. Introduction

β-Carotene (CAR) belongs to the carotenoid family. It is a natural lipophilic pigment composed of a polyene system with eleven conjugated double bonds and a β-ring at each end of the chain [1]. CAR is a versatile isoprenoid for human health. It is a precursor of vitamin A, and in humans, dietary β-carotene is cleaved to two molecules of all-trans-retinal by the action of β, β-carotene 15,15′–monooxygenase 1. Subsequently, retinal can be reduced into retinol by a retinal reductase or oxidized into retinoic acid by the action of retinal dehydrogenase [2]. CAR has a protective effect against oxidative stress, reduces the risk of Alzheimer’s disease [3], and is believed to prevent cancer [4], cardiovascular diseases [5], and type 2 diabetes [6]. Humans obtain CAR from a diet. It is found in carrots, green vegetables, pumpkins, and some other fruits and vegetables [7]. CAR is a beneficial ingredient for functional food and dietary supplements. However, it easily degrades under the influence of heat, light, and oxygen. Its insolubility in water and low chemical stability limits its application. Attention has to be paid to the development of proper CAR formulations and delivery systems. In general, the application of carotenoids in medicine and the food industry requires an increase in their hydrophilicity. In nature, there are only a few hydrophilic carotenoids. Abundantly natural occurring crocin and Na and K salts of bixin and norbixin are water-dispersible carotenoids [8]. For enhancing the hydrophilicity of carotenoids, they are mostly formulated with macromolecules. At present, emulsion-based systems, liposomes, and nanoparticles are largely known for CAR formulation [9,10,11]. For CAR entrapping, apoferritin nanocage, amylose, glycyrrhizic acid, arabinogalactan, and cyclodextrin (CD) are also used [10,12]. However, there is another way to obtain water-dispersible carotenoids, i.e., their chemical derivatization. As a successful example, tetracationic astaxanthin-lysine conjugate forms true monomolecular solutions. The critical aggregation concentration of that conjugate is rather high and equal to 2.18 mM [13]. The glycosylation of carotenoids or their conjugation with polyethyleneglycol also increases the hydrophilicity and water dispersibility [14].

Cyclodextrins are cyclic oligosaccharides composed of α-(1→4)-linked d-α-glucopyranose units. The cavity of CD is hydrophobic and suitable for entrapping a wide range of lipophilic “guest” molecules. α-, β- and γ-CD composed of 6, 7, and 8 glucose units are chemically stable, inexpensive, non-toxic and non-hygroscopic molecules. However, modified 2-hydroxypropyl-CD (HP-CD) is an alternative to traditional CD due to better solubility in water [15]. CDs are practically non-digestible by the enzymes in the upper gastrointestinal tract and are metabolized by the gut microflora. They are considered dietary fibers and have anti-obesity and anti-diabetic effects [16].

Usually, the preparation of CAR/HP-CD aggregate is performed by co-precipitation technique using ultrasound-assisted or conventional magnetic stirring approach [17]. There are a lot of variants for the implementation of the co-precipitation technique in scientific literature. Usually, the procedure lasts a long time, the mixture of carotenoid solution and the aqueous dispersion of CD aggregate is stirred for 16–48 h under a nitrogen atmosphere at 25–50 °C [18,19,20,21,22]. Previously, another procedure based on the addition of β-carotene solution in an organic solvent to the hot aqueous dispersion of CD aggregate and the following instant evaporation of the organic solvent was proposed [23]. The approach is attractive due to the shorter time of the preparation of CAR/HP-CD aggregate but is not fully examined. Therefore, the study is aimed to find the best conditions for preparing aqueous CAR/HP-CD aggregate dispersions using the above-mentioned procedure.

## 2. Results and Discussion

CAR/HP-CD aggregates were prepared at different HP-CD concentrations and reaction temperatures using various solvents for CAR dissolution. First, to estimate the amount of CAR entrapped into HP-CD aggregates, the best method for the extraction of CAR from CAR/HP-CD aggregates and transferring CAR into the organic phase must be chosen. Known published methods for the extraction of encapsulated carotenoids were analyzed and tested (Table 1). The best result in terms of extraction completeness was achieved using method 2. Therefore, this method was further used for the extraction of CAR and the estimation of its amount in the optimization process of the preparation of aqueous CAR/HP-CD aggregate dispersions.

**Figure 1:**
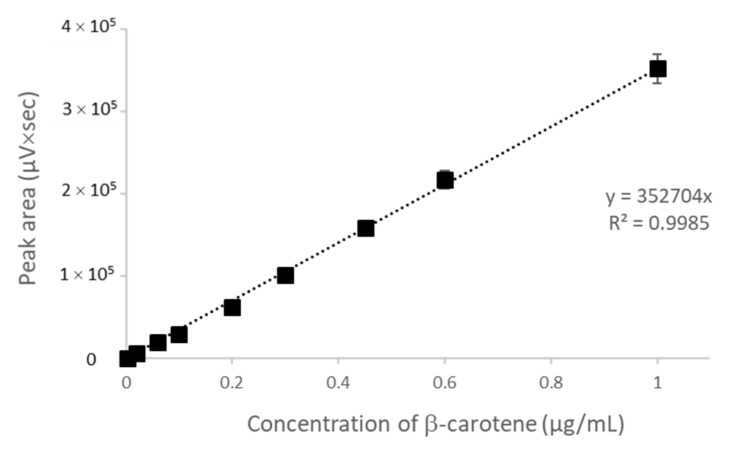
Calibration curve for β-carotene determination by HPLC using a C_18_ column.

**Figure 2:**
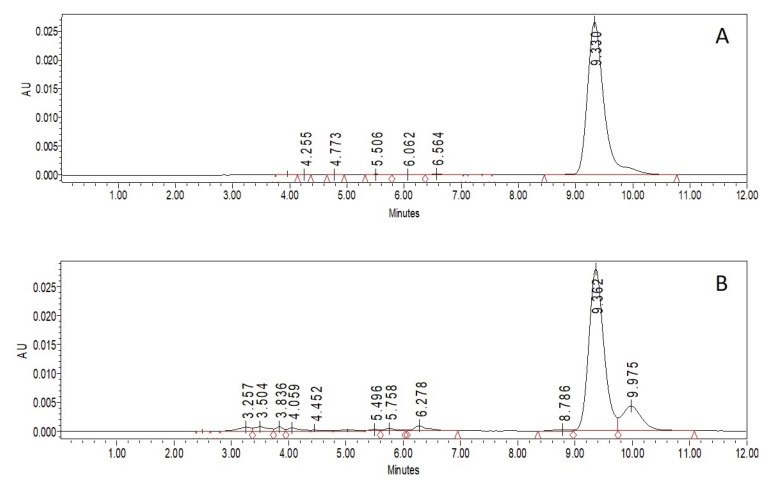
Chromatograms of pure CAR (**A**) and CAR extracted from CAR/HP-CD aggregates (**B**). CAR/HP-CD aggregates were prepared using 5% HP-CD in water, and CAR was dissolved in acetone.

CAR concentration was determined by HPLC with a C_18_ column using acetonitrile/dichloromethane/ethanol (70/20/10, *v*/*v*) and acetonitrile/methanol/tetrahydrofuran/20 mmol/L ammonium acetate in water (68/22/7/3, *v*/*v*) as a mobile phase [30,31]. Due to the better accuracy of the calibration curve (Figure 1), the second one was chosen. The retention time of the pure β-carotene and β-carotene extracted from the CAR/HP-CD aggregates was the same. On the chromatogram of the extract, a small peak with a retention time of 9.975 min appeared. Due to the entrapping process and β-carotene extraction from CAR/HP-CD aggregates, some amount of degradation products can be formed and result in the new peak on the chromatogram (Figure 2).

Since the preparation of CAR/HP-CD aggregates is based on dropwise adding of CAR dissolved in an organic solvent to a hot water dispersion of CD, four different solvents were tested for CAR dissolution, i.e., acetone, dichloromethane, hexane, and ethanol. The preparation of CAR/HP-CD aggregates was carried out at a water temperature that is higher than the boiling point of organic solvent to ensure its fast evaporation. As seen from Table 2, the highest content of entrapped CAR was found using acetone for its dissolution (Table 2). Dichloromethane and hexane are not miscible with water. It is plausible that it could be a reason for the low content of entrapped CAR.

In the case of ethanol, the high reaction temperature can have a negative effect increasing the degradation of CAR. As an example, the reaction rate constant of CAR thermal degradation in oil increases approximately 2.8 times with increasing reaction temperature from 75 to 85 °C [33]. On the other hand, CAR solubility in ethanol is very low compared to other solvents tested, and CAR practically remained dispersed. That could prevent the formation of CAR/HP-CD aggregates. The influence of temperature was evidenced by testing the preparation of CAR/HP-CD aggregates at a higher temperature using acetone as the best solvent for the reaction (Table 3). The entrapping process at 65 °C was about 5 and 12 times more effective than at 75 and 80 °C temperatures, respectively.

**Figure 3:**
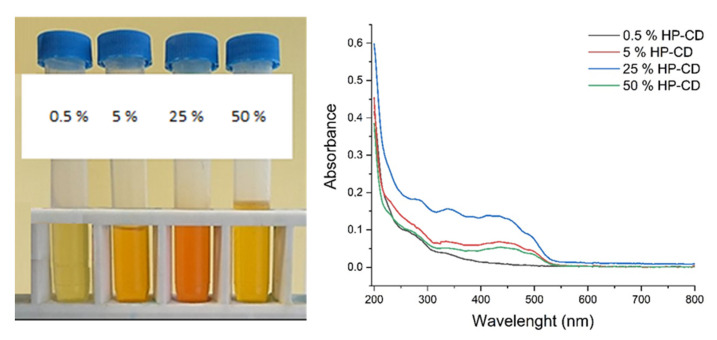
Samples of the CAR/HP-CD aggregates prepared at different concentrated aggregate dispersion of HP-CD and their VIS spectra.

Finally, the effect of HP-CD concentration on the formation of CAR/HP-CD aggregates was investigated. The highest amount of entrapped CAR was found using 25% aqueous dispersion of HP-CD (Table 3). The obtained dispersions of CAR/HP-CD aggregates and their spectra are presented in Figure 3. As seen, the use of 25% HP-CD resulted in the highest color intensity of CAR/HP-CD aggregate dispersions. The content of entrapped CAR decreases in the presence of 50% HP-CD. At present, it is well known that CD can spontaneously self-assemble in water solutions and form visible and subvisible aggregates. How and why CD aggregates is not fully clear. The phenomena of CD aggregation and the reliable methods for the analysis of aggregates are under investigation [34,35]. The aggregation of CD is a concentration-dependent process. The critical aggregation concentration value of 2-hydroxypropyl-β-cyclodextrin in water solution *c*_M_ ≈ 118 mg/mL [36]. Moreover, using molecular-dynamic calculations, Bikadi et al. demonstrated that the aggregation of CD is significant for their complexation behavior [37]. Therefore, the dependence of entrapped CAR content on the concentration of HP-CD can be also related to HP-CD behavior in water and the tendency for self-assembly.

**Figure 4:**
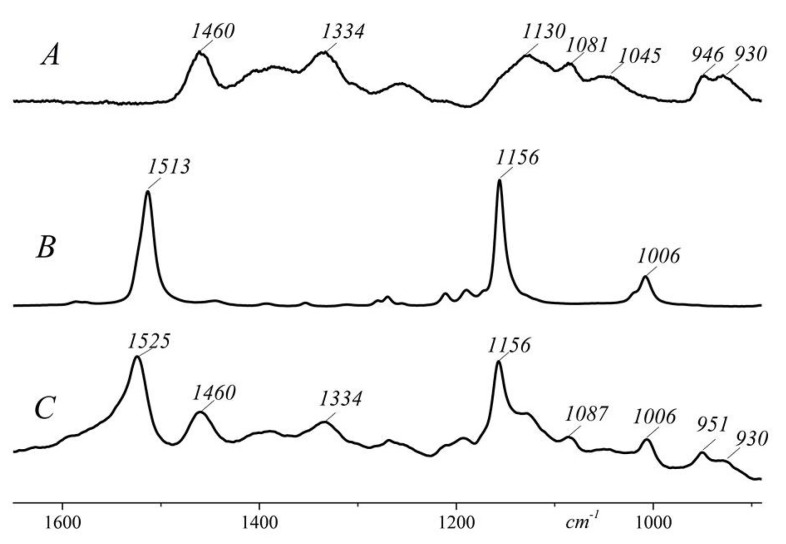
Raman spectra of HP-CD (**A**), pure CAR (**B**), and CAR/HP-CD aggregate (**C**).

The interaction of CAR with HP-CD was shown by Raman spectroscopy (Figure 4). Three bands of the pure CAR seen at 1513, 1156, and 1006 cm^−1^ are attributed to C=C, C-C stretching vibrations, and CH rocking vibrations of CH_3_ groups attached to the polyene chain coupled with C-C bonds [38]. The stretching modes of HP-CD produce relatively weak Raman signals. The region between 1200 and 1600 cm^−1^ contains the bands related to CH, CH_2,_ and COH deformation. The characteristic bands of CO stretching, C-C stretching and COC deformation modes, referring to the glycosidic bond are in the region between 1200 and 900 cm^−1^ [39]. The Raman spectrum of the CAR/HP-CD aggregate exhibits the complete spectroscopic vibrational pattern of the included non-polar guest except that the ν_1_ band is shifted from 1513 cm^−1^ to the higher wavenumbers 1525 cm^−1^. Due to the conformational changes induced by the CAR/HP-CD aggregate formation, an electronic conjugation degree decreases in the polyene chain [21]. Two other typical bands of β-carotene at 1156 and 1006 cm^−1^ were at the same positions after the CAR entrapping by the HP-CD [40]. In the Raman spectrum of the CAR/HP-CD aggregate, some bands related to the glycosidic bonds of HP-CD changed. Namely, the band at 946 cm^−1^ related to the symmetrical C-O-C stretching of pure HP-CD shifted to 951 cm^−1^, and the band at 1081 cm^−1^ attributed to C-O stretching of glycosidic bond shifted to 1087 cm^−1^ [41].

**Figure 5:**
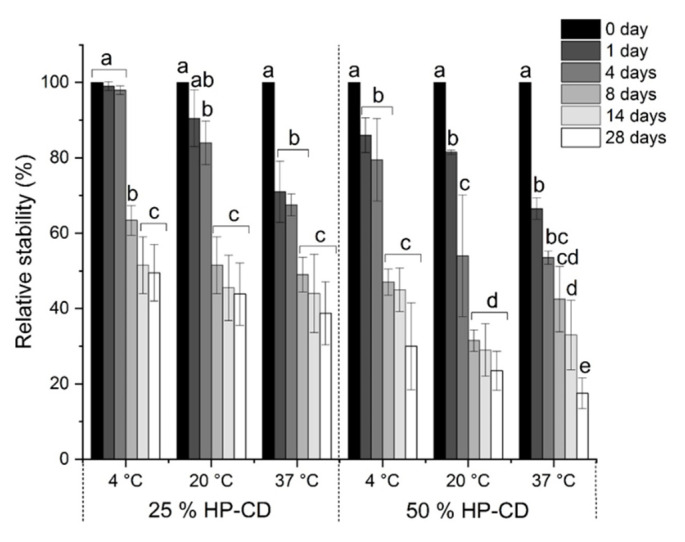
Relative stability of CAR in the CAR/HP-CD aggregates obtained using 25 and 50% HP-CD aqueous dispersions under the storage in the dark at different temperatures. Different letters indicate significant differences (*p* < 0.05) of CAR stability within each group of stability data.

The stability of entrapped CAR in the aggregates prepared using 25 and 50% HP-CD was investigated (Figure 5). As seen, the use of 50% HP-CD dispersion resulted in a sharp decrease of CAR content as compared to 25% HP-CD dispersion. It is obvious that at all temperature tested CAR is less stable when 50% HP-CD dispersion is used for the formation of CAR/HP-CD aggregates. It is plausible that at the high HP-CD concentration CAR adsorbs on the surface of HP-CD aggregates. Consequently, under storage at different temperatures CAR undergoes the degradation faster than CAR entrapped in the aggregates.

**Figure 6:**
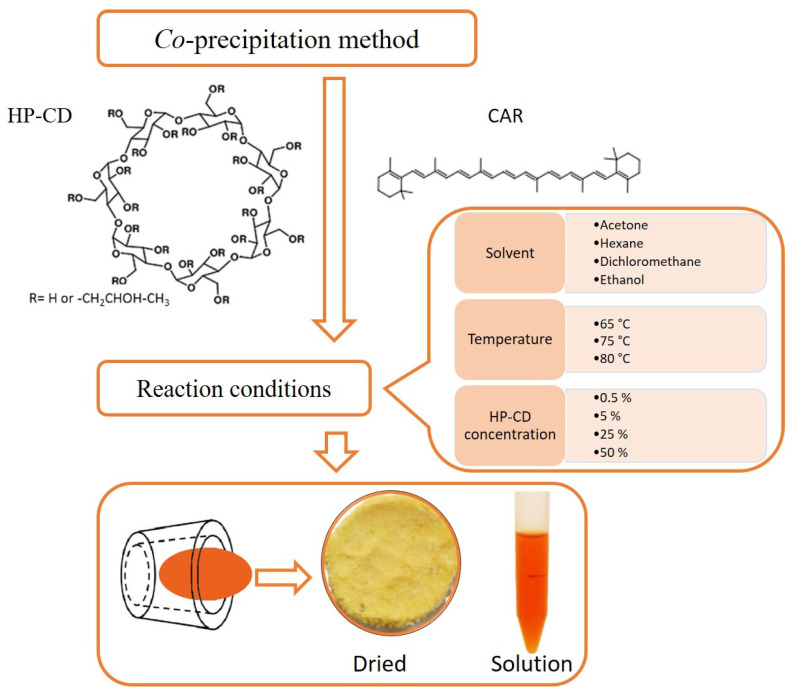
Schematic summary of the preparation of water-dispersible β-carotene/2-hydroxypropyl-β-cyclodextrin aggregates.

Figure 6 summarizes the work done and presented in the paper. The efficiency of entrapping is equal to 88% (25% HP-CD, acetone, 65 °C) and is comparable with the ones obtained by other aggregate formation procedures. As an example, stirring of β-cyclodextrin and β-carotene solution at 40 °C for 5 h resulted in an efficiency of 85% [42]. Lycopene-β-cyclodextrin aggregates were prepared by co-precipitation method using the stirring procedure for 24 h at 50 °C with an efficiency approximately of 72% [18]. CD is largely used for entrapping bioactive compounds due to their non-toxicity, stability, and low cost. Carotenoid-CD aggregates are formed to increase hydrophilicity and bioavailability of carotenoids and decrease their air-, light- and temperature sensitivity during the application and storage. For the preparation of aggregates, pure carotenoids or extracts from fruits and vegetables can be used [22,29,43,44]. On the other hand, the formation of guest-host aggregates results in a large mass portion of the carrier and a small mass portion of bioactive compounds. Having in mind the above-mentioned properties of CD and the fact that “empty” CD is used for the modification of the physical properties of foods [45], it cannot be considered as a drawback for CD application in the formulation of bioactive compounds. In conclusion, the optimized procedure presented in this study could be a choice for β-carotene/2-hydroxypropyl-β-cyclodextrin aggregates preparation.

## 3. Materials and Methods

### 3.1. Chemicals

β-Carotene (≥97.0%, UV) was purchased from Sigma-Aldrich (Darmstadt, Germany). 2-Hydroxypropyl-β-cyclodextrin (Cavasol W7 HP Pharma) was obtained from Ashland Industries Europe GmbH (Schaffhausen, Switzerland). Acetonitrile, dichloromethane, methanol, tetrahydrofuran, and ammonium acetate were purchased from Roth. n-Hexane, cyclohexane, and acetone were supplied from UAB “Eurochemicals” (Vilnius, Lithuania). Ethanol and dimethyl sulfoxide were obtained from Vilniaus degtine (Vilnius, Lithuania) and Honeywell Riedel-de-Haen, respectively.

### 3.2. Preparation of β-Carotene/2-Hydroxypropyl-β-Cyclodextrin Aggregate

For aggregate preparation, 1.75 g of HP-CD were dissolved in 7 mL of water. Separately, CAR was dissolved in acetone by stirring for 45 min to obtain the final concentration of 0.2 mg/mL. Subsequently, the aqueous dispersion of HP-CD at the concentration of 25% was heated to 65 °C, and 10 mL of CAR solution in acetone were added dropwise with rapid stirring for approximately 30 min. The reaction mixture was stirred until the complete acetone evaporation for approximately 15 min. The excess of CAR was removed using a polyether sulfone filter with 0.45 μm pore diameter. The obtained aqueous dispersion containing the CAR/HP-CD aggregates was analyzed and used for further experiments. Under the same procedure, CAR/HP-CD aggregates were prepared by using different solvents, temperatures, and HP-CD concentrations.

The efficiency of entrapping expressed as the ratio of the amount of CAR entrapped into HP-CD to the initial CAR amount used for the aggregate formation was calculated using the following equation:

EE (%) = ((W_0_ − W_1_)/W_0_) × 100 (1)

where W_0_ and W_1_ are the initial and unreacted amounts of CAR, respectively. W_1_ was determined gravimetrically.

### 3.3. Determination of Content of β-Carotene in CAR/HP-CD Aggregate

For the determination of entrapped CAR, 1 mL of the CAR/HP-CD dispersion was sonicated with 2 mL of ethanol and 3 mL of n-hexane (5 min sonication cycles, 15 s ON, 15 s OFF). The extraction process was repeated three times, obtained n-hexane fractions were combined, and the amount of extracted CAR was determined by using HPLC. n-Hexane fraction was evaporated and redissolved in the mobile phase of acetonitrile/methanol/tetrahydrofuran/20 mmol/L ammonium acetate in water (68/22/7/3, *v*/*v*). The chromatography was performed by using C_18_ column and isocratic elution at 1 mL/min. The absorbance was registered at 450 nm. The concentration of CAR was calculated using the following equation:

y = 352704x(2)

where y is a chromatographic peak area and x—CAR concentration, μg/mL.

### 3.4. Raman Spectroscopy Measurements

Raman spectra of the samples were recorded using a Perkin Elmer Raman Station 400F spectrometer using laser extraction at 785 nm. The spectra were recorded under the conditions as follows: a scan range of 300–3500 cm^−1^, laser power 100 W, accumulation time 15 s.

### 3.5. Relative Stability of Encapsulated β-Carotene

The water-dispersible CAR/HP-CD aggregates prepared by using 25 and 50% HP-CD were stored in the dark at 4, 20, and 37 °C temperatures. After 1, 4, 8, 14, and 28 days of storage, the samples were taken, and CAR was extracted from aggregates as described above. CAR concentration was determined in n-hexane fractions by UV/V is spectroscopy and calculated using the following formula [46]:

C = (Abs/(A^1%^ × d)) × DF × 10 (3)

where Abs is the absorbance of sample at 450 nm; A^1%^ is the mass extinction coefficient of β-carotene in n-hexane equal to 2590 g^−1^ L cm^−1^, d is the length of the cuvette (1 cm), C is the concentration of CAR in the sample (mg/mL) and DF is a dilution factor of sample.

Relative stability was calculated as follows:

Relative stability (%) = (C_0_/C_t_) × 100 (4)

where C_0_ and C_t_ are CAR content in aggregates at the initial moment and at the time t, respectively.

### 3.6. Statistical Analysis

Three parallel independent experiments were performed. Data are presented as mean ± standard deviation. One-way analysis of variance (ANOVA, *p* < 0.05) was used to compare the data and define the statistically significant result.

## 4. Conclusions

The procedure for the preparation of CAR/HP-CD aggregates by the co-precipitation method was optimized. The best conditions were found to be as follows: 25% concentration of 2-hydroxypropyl-β-cyclodextrin in water, 65 °C temperature, and acetone for β-carotene dissolution. The efficiency of entrapping was equal to 88%. CAR entrapped in the CAR/HP-CD aggregates prepared using 25% concentration of 2-hydroxypropyl-β-cyclodextrin in water exhibited higher stability compared to the ones prepared at 50% concentration of 2-hydroxypropyl-β-cyclodextrin in water.

## Figures and Tables

**Table 1:** Adopted methods of carotenoid extraction from the aggregates based on cyclodextrins.

No	Description of the Adopted Methods	References
1	1 mL of the aggregate water dispersion was mixed with 2 mL of ethanol and 3 mL of n-hexane and shaken for 15 min, 30 °C, 650 rpm. Subsequently, the mixture was ultrasonicated for 10 min and repeatedly shaken for 60 min, 30 °C, 650 rpm, or 120 min, 30 °C, 650 rpm.	[24]
2	1 mL of the aggregate water dispersion was mixed with 2 mL of ethanol and 3 mL of n-hexane and ultrasonicated for 2 min three times.	[24]
3	1 mL of the aggregates water dispersion was heated for 15 min at 70 °C, then mixed with 1 mL of cyclohexane for 3 min by shaking and ultrasonicated for 2 min.	[25]
4	1 mL of the aggregate water dispersion was heated for 15 min at 70 °C, then mixed with 1 mL of cyclohexane for 3 min by shaking.	[25]
5	1 mL of the aggregate water dispersion was mixed with 2 mL of dichloromethane for 1 min by shaking and centrifuged for 10 min, 290× *g*	[26]
6	1 mL of the aggregate water dispersion was mixed with 2 mL of acetone and 2 mL of n-hexane.	[27]
7	1 mL of the aggregate water dispersion was mixed with 9 mL of dimethyl sulfoxide for 3 min by shaking. Subsequently, the mixture was mixed with 1 mL of n-hexane and 4 mL of dichloromethane, shaken for 10 min, 25 °C, 650 rpm, and centrifuged for 10 min, 3000× *g*	[28]
8	The dry aggregates were dissolved in a mixture of ethanol and acetonitrile (5:1), ultrasonicated for 10 min, centrifuged for 5 min, 18 °C, 10,400× *g*	[29]

**Table 2:** The dependence of entrapped CAR content on CAR dissolution solvent.

Solvent	CAR Solubility, mg/L [32]	Temperature of Reaction, °C	Concentration of Entrapped CAR, µg/mL ^1,2^
Acetone	200	65	0.83
Dichloromethane	6000	47	0.02
Hexane	600	76	0.04
Ethanol	30	87	0.01

^1^ The content of entrapped β-carotene was calculated for 1 mL of aggregates at the concentration of 40 mg/mL. The CAR/HP-CD aggregates were prepared using 5% HP-CD in water. ^2^ The average mean from five experiments is presented.

**Table 3:** The dependence of entrapped CAR content on the reaction temperature and the concentration of HP-CD.

Temperature, °C	CAR Concentration in Aggregates, µg/mL ^1,2^			
	0.5% HP-CD	5 % HP-CD	25% HP-CD	50% HP-CD
65	0.11	0.83	2.21	0.75
75	-	0.17	-	-
80	-	0.07	-	-

^1^ The content of entrapped β-carotene was calculated for 1 mL of aggregates prepared using different concentrations of HP-CD. ^2^ The average mean from five experiments is presented.

## Data Availability

The data presented in this study are available on request from the corresponding author.

## Sample Availability

Samples of the compounds CAR, HP-CD and CAR/HP-CD are available from the authors.
